# Mamdani vs. Takagi–Sugeno Fuzzy Inference Systems in the Calibration of Continuous-Time Car-Following Models

**DOI:** 10.3390/s23218791

**Published:** 2023-10-28

**Authors:** Mădălin-Dorin Pop, Dan Pescaru, Mihai V. Micea

**Affiliations:** Computer and Information Technology Department, Politehnica University of Timisoara, 300223 Timisoara, Romania; madalin.pop@upt.ro (M.-D.P.); mihai.micea@cs.upt.ro (M.V.M.)

**Keywords:** intelligent transportation systems, fuzzy inference, car-following model, control systems, Mamdani, Takagi–Sugeno, online calibration

## Abstract

The transition to intelligent transportation systems (ITSs) is necessary to improve traffic flow in urban areas and reduce traffic congestion. Traffic modeling simplifies the understanding of the traffic paradigm and helps researchers to estimate traffic behavior and identify appropriate solutions for traffic control. One of the most used traffic models is the car-following model, which aims to control the movement of a vehicle based on the behavior of the vehicle ahead while ensuring collision avoidance. Differences between the simulated and observed model are present because the modeling process is affected by uncertainties. Furthermore, the measurement of traffic parameters also introduces uncertainties through measurement errors. To ensure that a simulation model fully replicates the observed model, it is necessary to have a calibration process that applies the appropriate compensation values to the simulation model parameters to reduce the differences compared to the observed model parameters. Fuzzy inference techniques proved their ability to solve uncertainties in continuous-time models. This article aims to provide a comparative analysis of the application of Mamdani and Takagi–Sugeno fuzzy inference systems (FISs) in the calibration of a continuous-time car-following model by proposing a methodology that allows for parallel data processing and the determination of the simulated model output resulting from the application of both fuzzy techniques. Evaluation of their impact on the follower vehicle considers the running distance and the dynamic safety distance based on the observed behavior of the leader vehicle. In this way, the identification of the appropriate compensation values to be applied to the input of the simulated model has a great impact on the development of autonomous driving solutions, where the real-time processing of sensor data has a crucial impact on establishing the car-following strategy while ensuring collision avoidance. This research performs a simulation experiment in Simulink (MATLAB R2023a, Natick, MA, USA: The MathWorks Inc.) and considers traffic data collected by inductive loops as parameters of the observed model. To emphasize the role of Mamdani and Takagi–Sugeno FISs, a noise injection is applied to the model parameters with the help of a band-limited white-noise Simulink block to simulate sensor measurement errors and errors introduced by the simulation process. A discussion based on performance evaluation follows the simulation experiment, and even though both techniques can be successfully applied in the calibration of the car-following models, the Takagi–Sugeno FIS provides more accurate compensation values, which leads to a closer behavior to the observed model.

## 1. Introduction

The importance of traffic simulation models has increased in recent years with the growing interest in driver assistance systems and autonomous driving systems. The development and calibration of such complex systems can greatly benefit from the use of simulations to reduce time and costs. Among other traffic simulation models, the continuous-time car-following model is particularly useful in the design and fine-tuning of various advanced driver assistance systems (ADASs) such as adaptive cruise control [1] and cooperative adaptive cruise control [2,3], or in developing autonomous driving technologies [4,5].

This paper aims to evaluate the performance of two well-known fuzzy inference techniques, Mamdani [6] and Takagi–Sugeno [7], adapted for car-following model calibration. To the best of our knowledge, this is the first study conducted to assess the performance of the two techniques used for the calibration of the car-following model. However, similar investigations have been carried out in many other domains where they serve to identify the appropriate fuzzy inference system (FIS) to solve specific uncertainty problems [8], as will be discussed in the next section.

The justification behind conducting this research also results from a literature search and analysis on the main scientific databases, such as ISI Web of Science (WoS), Scopus, and Google Scholar. The search was carried out between 2 June and 5 September. Table 1 shows the total work related to the use of FISs in the calibration of the car-following models and the specific query applied to each database. After removing the 13 duplicates, only 7 satisfy the search objective of reporting an innovative scientific contribution [9,10,11,12,13,14,15], the other articles being reviews or papers that do not fully satisfy the search objective. Unfortunately, none of the analyzed research developed a comparison between the behavioral analysis of the calibration of car-following models in relationships with the aforementioned inference techniques.

This research addresses a scientific literature gap related to a comparative analysis of the behavior of a car-following calibration system in the case of using Mamdani and Takagi–Sugeno FISs. To achieve this goal, this article has the following scientific contributions:

- Performs a literature search on the main scientific databases such as ISI WoS, Scopus, and Google Scholar and evaluates the results obtained based on their relevance to the comparative analysis of the application of Mamdani and Takagi–Sugeno FISs in the calibration of car-following models;
- Performs a literature review in two directions, one considering the approaches based on the application of the two fuzzy inference techniques in the calibration of car-following models, and another focusing on similar comparative investigations of these techniques in other fields of research;
- Proposes an original methodology to perform the comparison of Mamdani and Takagi–Sugeno FISs in the case of the calibration of car-following models by adapting their characteristics according to the needs of the modeling of this concept;
- Implements a three-parallel-simulation model in Simulink (MATLAB R2023a, Natick, MA, USA: The MathWorks Inc.) to reproduce the behavior of the observed car-following model and to calculate the compensation values to be applied to the input of the simulation model so that it replicates the behavior of the observed model; for this last purpose, two calibration models are implemented corresponding to the two fuzzy inference techniques;
- Validates the proposed methods for the calibration of the car-following model using real-world traffic data and provides a comparative evaluation in terms of the performance of the Mamdani and Takagi–Sugeno FISs in the context of the calibration of the car-following models;
- Identifies the limitations of this study and provides recommendations for future research.

## 2. Literature Review

This discussion of related work follows two directions. The first analyzes the applicability of fuzzy techniques in the implementation of the calibration process of car-following models, as identified during the literature analysis of the papers found according to the approach described in Section 1. The other direction consists of analyzing similar comparisons between Mamdani and Takagi–Sugeno FISs conducted in other domains, including also other topics found related to traffic monitoring and control.

Bennajeh and Ben Said [9] proposed a genetic algorithm for the calibration of their car-following model that uses an FIS in the lower level. This FIS was responsible for the anticipation of the velocities and drivers’ behaviors. Chakroborty and Kikuchi [10] transformed an FIS used for the calibration of a car-following model into an artificial neural network (ANN) to better model the stimulus–response relationships. Chen et al. [11] used camera calibration and video tracking technology in their comparison between a fuzzy-based car-following model and the Gazis–Herman–Rothery (GHR) model. Nadimi et al. [12] focused on collision avoidance and introduced a mixed index (MI) as a parameter to be calibrated considering car-following scenarios. This parameter was built as the output of an FIS having as input the time-to-collision (TTC) and post-encroachment time (PET). Pop et al. [13] developed a hybrid model for the calibration of car-following models that used a Kalman filter to eliminate the noises that affect the inputs of a Takagi–Sugeno FIS. This approach showed good results compared to a Kalman-filter-only approach for a standard car-following model, but also on a refined model that considered the lane change behavior [14] according to the evaluation of daytime and night-time traffic profiles. Sun et al. [15] built an FIS to calibrate car-following models based on two driving styles (i.e., aggressive and non-aggressive). The results showed good performance, especially in the case of an intelligent driver model (IDM).

However, the use of fuzzy-based solutions showed promising results in addressing the uncertainties in autonomous driving systems capable of ensuring collision avoidance. Pérez et al. [16] developed a fuzzy logic controller to adapt the velocity of the vehicle based on sensor data fusion retrieved with the help of radio frequency identification (RFID) technology. Terán et al. [17] used a Mamdani FIS in the development of a driver assistant responsible for alerting drivers in the case of traffic accidents based on the analysis of the risk map of the road accidents and vehicle telemetry. Fernandez et al. [8] proposed a classification system for driver behavior based on a Mamdani FIS that contains a knowledge base learned through a genetic algorithm. Ronquillo-Cana et al. [18] used a Mamdani FIS to classify driving styles in their approach that combined data obtained from sensors with the behavioral analysis of the drivers resulting from the application of the analytical hierarchy process (AHP) methodology on the questionnaires answered by these human subjects.

Similar comparative studies were conducted in various fields, such as optical [19] and wireless [20] networks, magnetic bearing system control [21], chaotic time series prediction [22], evaluation of user experience for specific applications [23], and streamflow prediction in the hydrological field [24]. Although Takagi–Sugeno usually showed better results, the research in the hydrological domain showed that the Mamdani FIS is more suitable. This is mainly explainable through the easier process of expressing human expertise with the Mamdani rules [24].

An increased number of articles that conducted comparisons between the fuzzy techniques analyzed in this study was observed in the field of power engineering. Phunpeng and Kerdphol [25] concluded that the Takagi–Sugeno FIS is more efficient in the adaptive inertia control of an islanded microgrid that considers the limitations and restrictions of thermal and wind generation. This technique was also appropriate for optimizing power extraction from photovoltaic solar systems in a simulation experiment considering various radiations [26]. The problem was formulated as a problem of maximum power point tracking (MPPT) with the goal of identifying the appropriate converter duty cycle to adapt to changes in environmental conditions, considering as input variables the slope of the voltage–power diagram and its changes [26,27]. Samavat et al. [26] also investigated the influence of the number of input membership functions, while Boutaybi et al. [27] analyzed the performance of the two fuzzy techniques compared to the perturb and observed method. The Mamdani FIS was also not considered suitable for optimizing the weight on bit during drilling operations in the research conducted by Khosravanian et al. [28] for two producing fields in Iran (i.e., Ahwaz oil field and Marun gas field formations).

However, the studies performed are not limited to the comparison of these two fuzzy techniques: they also investigated possible directions of optimization that can benefit in both cases. Bagis and Konar [29] applied the artificial bee colony algorithm to optimize the process of determining the parameters of the fuzzy models. Their experiment performed for a nonlinear system consisting of a microstrip antenna demonstrated a good contribution in terms of accuracy, reliability, and processing time.

The existing literature also offers some comparisons between Mamdani and Takagi–Sugeno FISs related to intelligent transportation systems (ITSs), except for the topic of car-following calibration. Saleh et al. [30] performed a comparison of these techniques in the design of a speed controller for a remote car, the analysis of the results showing better results in the case of the Takagi–Sugeno FIS. Another topic investigated is the prediction of traffic flow based on historical data [31], where the Takagi–Sugeno technique achieved faster processing times compared to Mamdani. However, the Mamdani technique proved to have a better performance, in terms of percentage error, in the prediction of traffic noise levels, as argued by Saleh et al. [32] based on the results of their simulation experiment.

## 3. Materials and Methods

**Figure 1:**
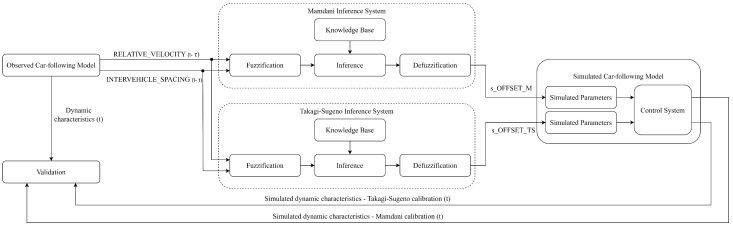
Methodological overview.

This research applies the methodology illustrated in Figure 1 to assess the impact of Mamdani and Takagi–Sugeno FISs in the calibration process of a continuous-time car-following model [33,34]. Since the measurement of sensor data and the simulation process are governed by uncertainty because of the errors than can be introduced in the simulated model, this problem is made suitable for implementation with an FIS [8,13,14]. In this way, the identification of the right compensation value that should be applied to the inputs of the simulated car-following model to counteract the aforementioned errors operates with a metric of fuzzy logic called the degree of membership [35]. This evaluation implies the existence of an observed model, a simulated model, and a validation system responsible for the identification of how faithfully the simulation model replicates the behavior of a real-world system (i.e., observed model).

A simulated car-following model has as main components a subsystem designed for parameters handling and a subsystem responsible for the control strategy of the vehicle behind (i.e., FV—follower vehicle) based on the observed behavior of the vehicle moving ahead (i.e., LV—leader vehicle), while ensuring collision avoidance [36]. In this research, the analysis of the calibration process requires the design and implementation of two calibration models, one using the Mamdani and the other the Takagi–Sugeno inference engines, in establishing the corresponding compensation values s_OFFSET_M and s_OFFSET_TS to be applied to the simulated running distance of FV $\left(x_{4}\right)$.

The calibration system uses RELATIVE_VELOCITY and INTERVEHICLE_SPACING at time t−τ as input variables in the alignment of the simulated value of the running distance of the FV with the value corresponding to the observed model at time *t* for both cases of inference engines. The validation system compares the dynamic characteristics of the observed system at time *t* with the simulated values obtained at time *t* after applying the compensation values according to the output of the calibration system for both cases of inference engines. As shown in Figure 1, the simulated system maintains the same control strategy [33,34] while separately handling the parameters for both cases evaluated in this article.

In the following subsections, this article presents in detail the characteristics of each of the systems involved in this research. Furthermore, an experimental case study based on real traffic data is chosen to validate this research. All the following information allows other researchers to replicate this study and facilitates future developments.

### 3.1. Continuous-Time Car-Following Modeling and Calibration

The representation of traffic phenomena at microscopic level ensures a better granularity in identifying the root cause of traffic congestion and simplifies the identification of appropriate measures to improve the traffic control systems. According to Yin et al. [37], this road traffic model consists of four levels of representation, such as crossroads configuration, links, lane choice, and car following. The last level mentioned considers a single lane of traffic where the vehicles are moving in a chain and describes the control strategy of the vehicle behind (FV—follower vehicle) based on the observed behavior of the vehicle moving ahead (LV—leader vehicle) [36].

**Figure 2:**
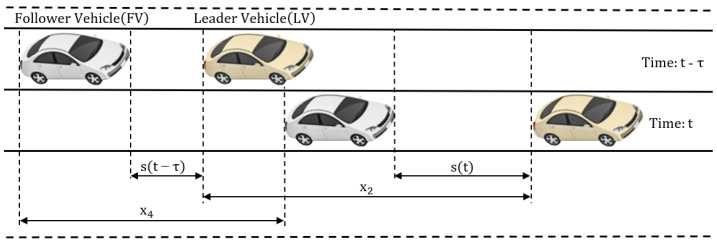
Dynamic characteristics of the car-following model [14].

Figure 2 illustrates the concept of car following. This concept defines the movement in a traffic lane based on pairs of vehicles consisting of an LV and FV. The dynamic interaction between them should consider the behavior of the LV, the FV permanently adapting its control strategy to avoid collisions and maintain a safety distance during movement. Figure 2 depicts the dynamic characteristics associated with the concept of car following, such as the running distances of LV ($x_{2}$) and FV ($x_{4}$) and the evolution of the dynamic distance between the vehicles from s(t) to s(t−τ) during a period of time equal to τ. These distances are directly influenced by driver behavior, and an update in the velocity of the LV should be immediately observed by the FV and an acceleration or deceleration should be applied accordingly. In the case of autonomous driven vehicles, the observed behavior of the LV results from the sensor data processing, so the existence of high measurement errors can lead to traffic collision between the FV and LV.

The state-space representation of the car-following model in continuous time and without time delay in Equation (1) uses the dynamic characteristics illustrated in Figure 2 and also new characteristics such as the velocity $\left(x_{1}\right)$ and acceleration $\left(u_{1}\right)$ of the LV, the velocity $\left(x_{3}\right)$ and acceleration $\left(u_{2}\right)$ of the FV, and the standard safety distance *S* [33,34].

(1) $$\left\{\begin{array}{c} \left[\begin{array}{c} {\dot{x}}_{1}\\ {\dot{x}}_{2}\\ {\dot{x}}_{3}\\ {\dot{x}}_{4} \end{array}\right]=\left[\begin{array}{cccc} 0 & 0 & 0 & 0\\ 1 & 0 & 0 & 0\\ 0 & 0 & 0 & 0\\ 0 & 0 & 1 & 0 \end{array}\right]\cdot \left[\begin{array}{c} x_{1}\\ x_{2}\\ x_{3}\\ x_{4} \end{array}\right]+\left[\begin{array}{cc} 1 & 0\\ 0 & 0\\ 0 & 1\\ 0 & 0 \end{array}\right]\cdot \left[\begin{array}{c} u_{1}\\ u_{2} \end{array}\right]\\ \;y=\left[\begin{array}{cccc} 0 & -1 & 0 & 1 \end{array}\right]\cdot \left[\begin{array}{c} x_{1}\\ x_{2}\\ x_{3}\\ x_{4} \end{array}\right]+S \end{array}\right.$$

The calculation of the standard safety distance *S* applies the average length of the vehicle for passenger cars (L=4.50 m) according to Equation (2). The reason behind this calculation is to avoid collisions between the FV and LV during movement.

(2) $$S=L\cdot (1+\frac{x_{3}}{16.10})$$

The purpose of the calibration process is to identify the compensation values that will be applied to the parameters of the simulated model to ensure that it fully reproduces the behavior of the observed system. Equation (3) is the continuous-time state-space representation of the calibration model corresponding to a car-following scenario [38]. This illustrates the parameters to be calibrated and the corresponding compensation values such as $\gamma_{1}\left(t\right)$ for the velocity of the LV, $\gamma_{3}\left(t\right)$ for the velocity of the FV, and $\gamma_{s}\left(t\right)$ for the dynamic distance between vehicles.

(3) $$\left\{\begin{array}{c} {\dot{x}}_{1}\left(t\right)=x_{1}\left(t\right)+\gamma_{1}\left(t\right)\\ {\dot{x}}_{3}\left(t\right)=x_{3}\left(t\right)+\gamma_{3}\left(t\right)\\ \dot{s}\left(t\right)=s\left(t\right)+\left[x_{1}\left(t\right)-x_{3}\left(t\right)\right]\cdot T+\gamma_{s}\left(t\right) \end{array}\right.$$

Furthermore, the calibration considers the measurement errors that are already part of the observed parameters as defined in (4) considering a space length *T* [38]. The notation used has the following significance: $\zeta_{1}\left(t\right)$ is the measurement error of the LV velocity, $\zeta_{3}\left(t\right)$ is the measurement error of the FV velocity, and $\zeta_{s}\left(t\right)$ represents the measurement error of the dynamic distance between vehicles.

(4) $$\left\{\begin{array}{c} {\dot{x}}_{1}^{obs}\left(t\right)=x_{1}\left(t\right)+\zeta_{1}\left(t\right)\\ {\dot{x}}_{3}^{obs}\left(t\right)=x_{3}\left(t\right)+\zeta_{3}\left(t\right)\\ {\dot{s}}^{obs}\left(t\right)=s\left(t\right)+\zeta_{s}\left(t\right) \end{array}\right.$$

### 3.2. Fuzzy-Based Calibration of Continuous-Time Car-Following Models

**Figure 3:**
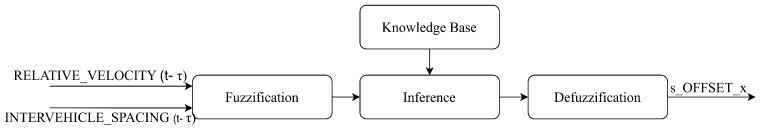
The main components of a fuzzy inference system (FIS).

Implementing an FIS (Figure 3) requires the following key components: fuzzification, knowledge base, inference, and defuzzification. The role of each component is explained further in relation to the topic of this research. Fuzzification is responsible for converting the numerical input values (i.e., the observed relative velocity RELATIVE_VELOCITY and the spacing INTERVEHICLE_SPACING between the LV and FV at time t−τ) into linguistic variables. The names chosen for the linguistic variables are similar to those existing in other similar studies [21,27,39,40], such as negative big (NB), negative small (NS), zero (ZE), positive small (PS), and positive big (PB). The knowledge base contains the rules expressed as “IF THEN” conditions that define the logical relations between the inputs RELATIVE_VELOCITY and INTERVEHICLE_SPACING and the output s_OFFSET_x, where x={M;TS} is defined separately for Mamdani (Section 3.2.2) and Takagi–Sugeno FISs (Section 3.2.3). These two components (i.e., fuzzification and knowledge base) are the same for both the Mamdani and Takagi–Sugeno FISs and are further described in Section 3.2.1, specially designated for the common configuration.

Inference represents the decision-making process for the assessment of output values based on the degree of membership of the inputs according to the content of the knowledge base. Details related to each inference method are available in the specific sections (Section 3.2.2 and Section 3.2.3).

Defuzzification converts the fuzzy sets [35] into a numerical value s_OFFSET_x, where x={M;TS} represents the dynamic distance between the FV and LV that is used as a compensation value to update, at time *t*, the simulated parameter of the running distance $x_{4}$ corresponding to the FV. The scientific literature uses the term “crisp value” when referring to this output as a numerical value of an FIS.

#### 3.2.1. Common Configuration

The fuzzification step is similar to the approach of Li and Zhu [39], who developed a new control strategy for car-following scenarios. The main difference arises from the choice of a minimum distance between the LV and FV (of S=4.50 m) because this article uses real-world traffic data for validation instead of simulation-generated data as in the research by Li and Zhu [39] and, consequently, considers Equation (2) that, according to Khodayari et al. [34] and Pan and Zheng [33], avoids collisions during a car-following scenario. This influences the definition of both input linguistic variables, with the detailed intervals shown in Table 2. In all cases, the significance of the membership functions is defined at the beginning of Section 3.2. RELATIVE_VELOCITY is expressed in m/s and the distance INTERVEHICLE_SPACING is expressed in meters.

**Figure 4:**
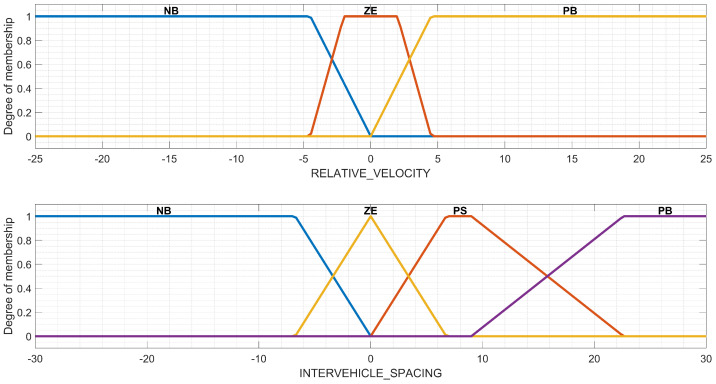
Membership functions of the inputs of the fuzzy inference systems (FISs).

Figure 4 shows a visual representation of the inputs as a result of the implementation of the FIS in MATLAB R2023a, Natick, MA, USA: The MathWorks Inc. using the *Fuzzy Logic Toolbox*. The expression of the linguistic variable called RELATIVE_VELOCITY can have the membership values in one of the fuzzy sets NB, ZE, or PB. The choice of these three fuzzy sets is related to the approach of Li and Zhu [39] and is strengthened by the need for a fast decision-making process to identify the right reaction in the control strategy of the FV based on the observed behavior of the LV. The reading of sensor data can introduce delays, and, consequently, an erroneous value received from the sensor network could lead to an unwanted collision between the FV and LV. In this way, it is not feasible to define the fuzzy sets NS and PS for this variable because their use cannot guarantee collision avoidance. For the variable INTERVEHICLE_SPACING, the fuzzy sets NB, ZE, PS, and PB can be defined because they represent the ideal spacing between the FV and LV as they result from the observed model and there is no need for a real-time read of the sensor data. However, the use of NS is not necessary because a correlation with the measurement of sensor data can lead in some cases to collisions; consequently, the choice was to adopt a prudential attitude for this variable also. The degree of membership for each input variable complies with the definition from Table 2.

Table 3 contains the knowledge base associated with Mamdani and Takagi–Sugeno FISs. Consequently, this matrix form expresses the mapping between the output s_OFFSET_M and s_OFFSET_TS, respectively, of the inference engine based on the fuzzified inputs RELATIVE_VELOCITY and INTERVEHICLE_SPACING.

#### 3.2.2. Mamdani

Table 4 presents the membership functions for the output of the Mamdani FIS; the significance is the same as defined at the beginning of Section 3.2, and s_OFFSET_M is expressed in meters. The reason behind choosing the range as [−25.50;25.50] for the output s_OFFSET_M is that the implemented car-following model by default sums the standard safety distance S in the FV control strategy as defined in Equation (1). Following this approach, the range chosen for s_OFFSET_M corresponds to the range set for INTERVEHICLE_SPACING.

**Figure 5:**
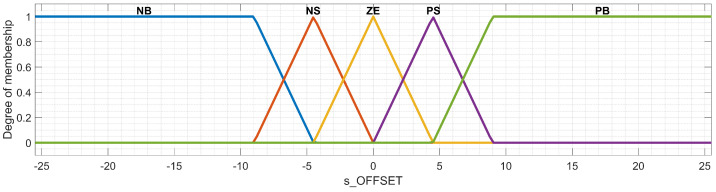
Membership functions of the output of the Mamdani fuzzy inference system (FIS).

A graphical representation of the membership functions associated with the output s_OFFSET_M is available in Figure 5. The method chosen for defuzzification in the case of the Mamdani FIS uses the center of gravity technique (i.e., centroid); this method is applied in many other works [20,27,28,41,42]. According to this defuzzification technique, a crisp output *Z* calculated for continuous values uses the curve of the scaled output membership function μ(x), as expressed in Equation (5) [29,43].

(5) $$Z=\frac{\int_{\mu }\mu \left(x\right)x\;dx}{\int_{\mu }\mu \left(x\right)\;dx}$$

**Figure 6:**
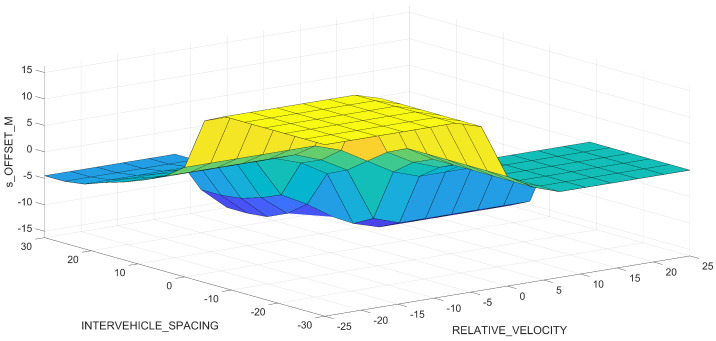
Surface visualization of the Mamdani fuzzy inference system (FIS).

Figure 6 illustrates a three-dimensional view of the relationship between the inputs (i.e., RELATIVE_VELOCITY and INTERVEHICLE_SPACING) and the output (i.e., s_OFFSET_M) of the Mamdani FIS. The flat region on the right shows a constant behavior in the output of the degree of membership of the output fuzzy set. The yellow area indicates the area with the most activated output fuzzy set associated with the input values of RELATIVE_VELOCITY and INTERVEHICLE_SPACING. The large flat area associated with this yellow area shows that the activation of it has a constant behavior.

#### 3.2.3. Takagi–Sugeno

**Figure 7:**
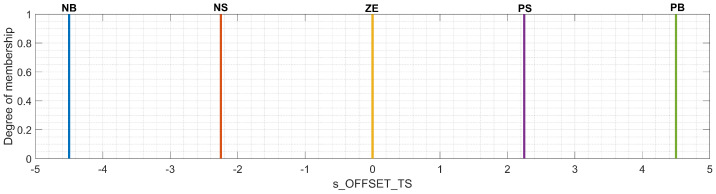
Membership functions of the output of the Takagi–Sugeno fuzzy inference system (FIS).

Figure 7 shows a visual representation of the configuration set for the membership functions associated with the output s_OFFSET_TS. The significance of the membership functions is kept as at the beginning of Section 3.2. Compared to the Mamdani FIS, the Takagi–Sugeno technique uses crisp values to represent the output. The choice of values corresponding to the membership functions is argued through a worst-case scenario where an additional value of PB equal to the standard safety distance S=4.50 m should be applied as a compensation value to the simulated running distance of the FV $x_{3}$ to avoid collisions with the LV. Otherwise, the application of a compensation value corresponding to NB contributes to the stability of the simulated model and directly influences the behavior of other pairs of (LV, FV) moving in a chain inside the same traffic lane, behind the pair of (LV, FV) targeted for calibration.

The defuzzification of the Takagi–Sugeno FIS uses the weighted average technique for the output and considers the constant values defined for the five membership functions represented in Figure 7. Consequently, Equation (6) expresses the calculation of the crisp output *Z* as a weighted average of the total clipped singletons [30,31].

(6) $$Z=\frac{\sum_{i=1}^{n}\mu \left(z_{i}\right)\cdot z_{i}}{\sum_{i=1}^{n}\mu \left(z_{i}\right)}$$

**Figure 8:**
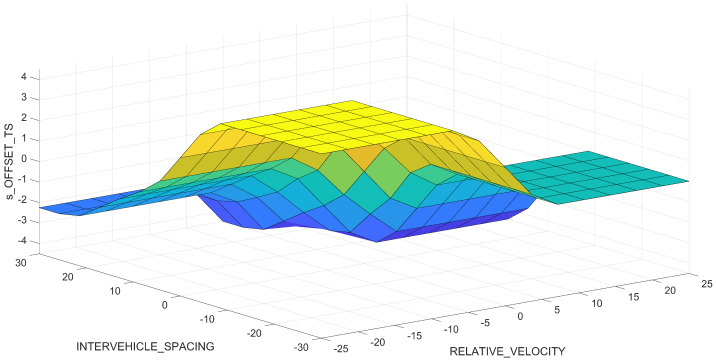
Surface visualization of the Takagi–Sugeno fuzzy inference system (FIS).

The surface diagram describing the three-dimensional relationship between the inputs RELATIVE_VELOCITY and INTERVEHICLE_SPACING, respectively, and the output s_OFFSET_TS of the Takagi–Sugeno FIS, is available in Figure 8.

### 3.3. Experimental Case Study—Simulation Model

**Figure 9:**
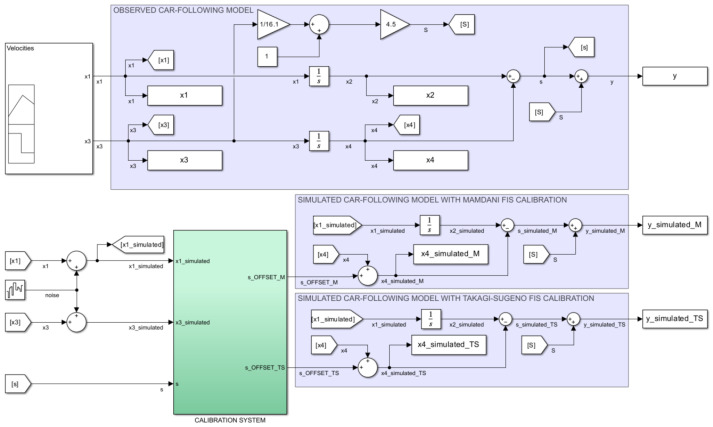
Simulation model: implementation of the car-following model using Simulink (MATLAB R2023a, Natick, MA, USA: The MathWorks Inc.).

The simulation experiment uses Simulink (MATLAB R2023a, Natick, MA, USA: The MathWorks Inc.) to implement the car-following model (Figure 9). Compared to the approach of Khodayari et al. [34] and Pan and Zheng [33], which represents the basis of the model used in this research, this simulation model was modified to introduce parallel calculations for the running distance $x_{4}$ of the FV and for the dynamic safety distance *y* to cover the needs for the visualization of the simulated behavior of the car-following model in the presence of Mamdani and Takagi–Sugeno FISs, respectively. This allows for an easier comparison to the observed model, which is considered as the ideal model (i.e., free of measurement errors). Consequently, Figure 9 illustrates three implementations of the continuous-time car-following model [33,34] marked and referred accordingly in the picture to comply with the methodology described in Figure 1. The observed car following uses as input the velocities of the LV $\left(x_{1}\right)$ and FV $\left(x_{3}\right)$ and calculates the output consisting of the dynamic safety distance (y) according to Equations (1) and (2).

Following the methodology illustrated in Figure 1, a calibration system is introduced to update the parameters of the simulated model by applying the right compensation values so that this model can fully replicate the behavior of the observed model. Since there are two fuzzy techniques to be evaluated within this research (i.e., Mamdani and Takagi–Sugeno), Figure 9 shows its parallel implementation and emphasizes the application of the determined compensation values (i.e., s_OFFSET_x,x={M;TS}) to the corresponding simulated car-following models. To observe the behavior of the two fuzzy-based calibration models, measurement and simulation errors are simulated by noise injection using the Simulink block entitled Band-Limited White Noise with the following characteristics: noise power = 0.10, sample time = 0.10, and seed = 23,341 [13]. The noise produced is a function of normally distributed random numbers and includes both measurement and simulated model errors.

**Figure 10:**
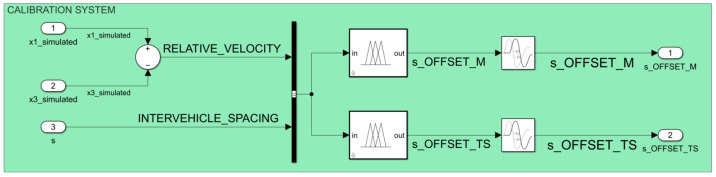
Simulation model: implementation of the comparative calibration system using Simulink (MATLAB R2023a, Natick, MA, USA: The MathWorks Inc.).

The detailed view of the calibration subsystem (Figure 10 shows that it contains a parallel implementation of Mamdani and Takagi–Sugeno FISs as shown in the conceptual model (Figure 1). The development of these FISs uses the *Fuzzy Logic Toolbox* available in MATLAB R2023a (Natick, MA, USA: The MathWorks Inc.) and complies with the definition of membership functions previously presented in this methodology. This implementation uses the notation INTERVEHICLE_SPACING to rename the dynamic distance *s* (i.e., the ideal distance between the FV and LV as it results from the observed model without the application of the standard safety distance S) so that it can comply with the fuzzy theory that uses the notation with easily interpreted significance [24] and is according to the notation adopted in the definition of membership functions. The use of the Simulink block entitled *Transport Delay* immediately after the output of the *Fuzzy Logic Controller* blocks aims to cover the processing and response time of these units and, consequently, to ensure that the proposed calibration model fits to real-time processing.

**Figure 11:**
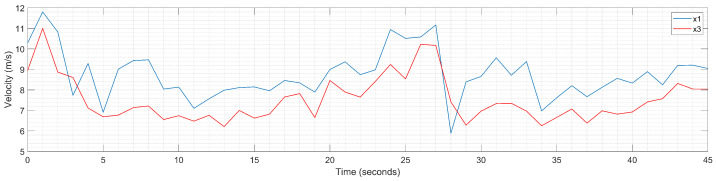
Simulation model: velocity profiles for the leader vehicle (LV) and the follower vehicle (FV) used as input for the implementation of the car-following model using Simulink (MATLAB R2023a, Natick, MA, USA: The MathWorks Inc.) [13].

The validation of the calibration models uses real traffic data from the local center for traffic monitoring and control in Timișoara city, Romania. The input data consist of velocity profiles (Figure 11) for the LV and FV, collected by inductive loop detectors from urban infrastructure during August 2019; the corresponding section of the road is located between two crowded crossroads (i.e., Liviu Rebreanu–Calea Șagului and Liviu Rebreanu–Gheorghe Ranetti) [13].

### 3.4. Performance Evaluation

Performance analysis considers the calibration time of Mamdani and Takagi–Sugeno FISs as the main method of evaluation; this metric is also observable based on a visual analysis of the obtained results.

To obtain a better overview in terms of performance, this research also uses the same methodology as in other similar studies [9,11,31,44,45,46]. These evaluation metrics are the mean absolute error (MAE) and root mean square error (RMSE). Equations (7) and (8) express the calculation for these metrics used for performance evaluation, where $Y_{i}^{obs}$ and $Y_{i}^{sim}$ are the *i*-th recordings of observed and simulated values from a total of *N* recorded data.

(7) $$MAE=\frac{1}{N}\cdot \sum_{i=1}^{N}\left|Y_{i}^{obs}-Y_{i}^{sim}\right|$$

(8) $$RMSE=\sqrt{\frac{1}{N}\cdot \sum_{i=1}^{N}{\left(Y_{i}^{obs}-Y_{i}^{sim}\right)}^{2}}$$

The calculation of these metrics for the running distance $x_{4}$ of the FV and for the dynamic safety distance *y* is available in Section 5.

## 4. Results

**Figure 12:**
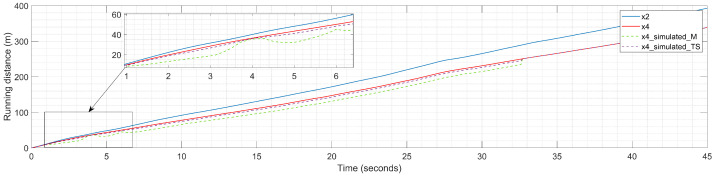
Experimental results: running distances for leader vehicle (LV) and follower vehicle (FV) during calibration process.

The simulation results (Figure 12) show that both the Mamdani and Takagi–Sugeno FISs succeeded in calibrating the car-following model used in this simulation experiment. A visual analysis of the results obtained demonstrates that collision avoidance is ensured in both cases, something recognized by the absence of intersection points between the running distances of the LV ($x_{2}$) and FV ($x_{4}$).

The Takagi–Sugeno FIS behaves better than the Mamdani FIS in identifying the time-varying appropriate compensation values that, applied to the simulated running distance of the FV ($x_{4}\_simulated\_TS$), are close to the observed value $x_{4}$ compared to the simulated running distance of the FV ($x_{4}\_simulated\_M$) obtained in the case of the Mamdani FIS. A detailed overview of the calibration process also demonstrates a more stable calibration process in the case of the Takagi–Sugeno FIS, while the Mamdani FIS needs more time to achieve a stable behavior, and some perturbations appear at the beginning. The perturbations are strongly related to the definition of the output membership functions as fuzzified values in the case of the Mamdani FIS, while the definition of the output membership functions for the Takagi–Sugeno FIS as a crisp constant value offers a more predictable support in identifying the appropriate compensation values.

At the same time, between achieving stable behavior and successfully calibrating the car-following model, the Mamdani FIS introduces a higher uniform increase in computation error that leads to scaled-down values of the running distance of the FV ($x_{4}\_simulated\_M$) compared to the values obtained in the case of the Takagi–Sugeno FIS ($x_{4}\_simulated\_TS$). However, these scaled-down values also appear in the case of the Takagi–Sugeno FIS compared to the observed value of the running distance of the FV ($x_{4}$), but are smaller; a complete overview of this impact is provided in Section 5.

**Figure 13:**
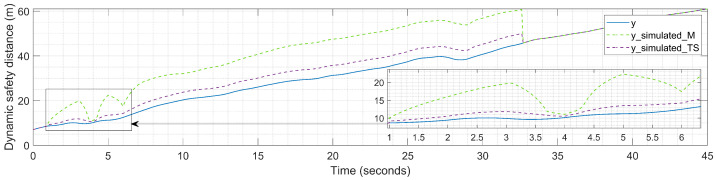
Experimental results: dynamic safety distance during calibration process.

The effect of the two implemented calibration methods is also visible in the output of the car-following model consisting of the dynamic safety distance *y*, as illustrated in Figure 13. Here, a better overview of the impact of the differences between the two fuzzy techniques is provided. The application of the standard safety distance *S* according to Equation (2) makes it more visible that the Takagi–Sugeno FIS achieves better performance compared to the Mamdani FIS. Based on several computations, the effect of the scaled-down simulated values of the FV running distance is reflected in the scaled-up simulated values of the dynamic safety distance *y*.

## 5. Discussion

The results obtained after performing the simulation experiment demonstrate that both fuzzy techniques analyzed in this article are suitable for applications responsible for the online calibration of car-following models and fit to real-time processing. This statement is strengthened by the existing literature because the uncertainties related to the calibration process of car-following models require the implementation of applications where interpretability and linguistic descriptions are needed (e.g., decision support systems, expert systems, etc.) [6,24,47]. At the same time, the Takagi–Sugeno FIS is considered suitable for applications (e.g., control systems, function approximation, etc.) that need crisp numerical outputs [7,26,27].

However, there are differences in terms of computational efficiency; the existence of perturbations and higher scale-down simulated values of the FV running distance before the system achieves the calibrated state are explained by the existing literature. The Mamdani FIS identifies fuzzy output values through the aggregation of multiple fuzzy rule outputs [6,24,26,27], like the center of gravity technique applied in this research, while each fuzzy rule contributes a weighted linear function to the overall output in the case of the Takagi–Sugeno FIS [7,26,27].

Table 5 presents the values of the performance metrics used in this research, calculated based on the obtained values of the simulated running distance $x_{4}$ of the FV corresponding to both fuzzy techniques used in the calibration of the car-following models. MAE expresses the average magnitude of errors between the observed and simulated values and shows that the Takagi–Sugeno FIS is able to identify the most appropriate compensation values, leading to closer values of the simulated FV running distance to the observed model compared to the values obtained based on the Mamdani FIS. Furthermore, analysis of the RMSE values indicates that the Takagi–Sugeno FIS has better predictive accuracy compared to the Mamdani FIS.

Table 6 shows the values calculated for the performance metrics used in this research (i.e., MAE and RMSE) based on the dynamic safety distance *y* to validate the application of Mamdani and Takagi–Sugeno FISs in the calibration of a continuous-time car-following model. Similar to the evaluation of these metrics in the case of running distance $x_{4}$, the Takagi–Sugeno FIS shows better performance demonstrated by reduced simulation errors. The efficiency of both techniques is visible because the differences in the values obtained for these indicators are similar to those obtained in Table 5, even if here the simulation and measurement errors related to the velocity of the LV $\left(x_{2}\_simulated\right)$ are additionally considered. The application of the standard safety distance *S* in the calculation of *y* has no effect on influencing the performance of the two fuzzy techniques because it applies to all the time-varying processed parameters and is a constant value that is provided by the car-following strategy and cannot be measured.

Thus, even if both fuzzy techniques can be successfully applied for the calibration of car-following models, the Takagi–Sugeno FIS is characterized by more accurate results, the computational efficiency being ensured by the use of constant crisp values in the definition of the membership functions and the application of the weighted average technique in the determination of the FIS output.

## 6. Conclusions

This article addresses a gap in the scientific literature consisting of the absence of a comparative analysis between the application of Mamdani and Takagi–Sugeno FISs for the calibration of car-following models. The proposed methodology for performing this comparison consists of defining the membership functions of both FISs considering the standard safety distance that should be applied in the FV control strategy to avoid collisions during travel. Although the input membership functions and the fuzzy rules are the same, the differences arise from the definition of the output membership functions (i.e., the Mamdani FIS contains fuzzified values and the Takagi–Sugeno FIS has constant crisp values).

A simulation experiment performed in Simulink (MATLAB R2023a, Natick, MA, USA: The MathWorks Inc.) shows that both FISs succeeded in calibrating the car-following model. However, the Takagi–Sugeno FIS obtains better results in terms of performance according to the evaluation of performance metrics represented by MAE and RMSE, widely used for evaluation in related work. The impact of the computation errors that lead to scaled-down values of the running distance of the FV is also observable in both cases, but the differences are smaller in the case of the Takagi–Sugeno FIS.

Furthermore, the Takagi–Sugeno FIS is suitable for use in the calibration process of car-following models based on the stability process of the identification of the time-varying compensation values, whereas the approach using the Mamdani FIS is affected by perturbations. The source of these perturbations is strongly connected to the definition of output membership functions: the higher surface of values covered in the case of the Mamdani FIS can introduce erroneous compensation values, while the Takagi–Sugeno FIS offers a more stable behavior because of the definition of output membership functions as crisp constant values.

However, this research also has some limitations that can be addressed in future research. This results from the intervals chosen for the membership functions and also from the type of function chosen. The particularity of the continuous-time car-following model used in this research can influence the behavior of the calibration method and can impact the performance of the FIS. The weaknesses of this research are also related to the use of simulated fault injection to represent the errors from the measurement of sensor data and those introduced by the simulation process, the configuration of the Simulink Band-Limited White Noise block also having influence on the calibration process.

## 7. Future Research

Future research can perform comparative evaluations of these two fuzzy techniques in the presence of additional control mechanisms such as genetic algorithms or neural networks. Also, it is worth analyzing how Mamdani and Takagi–Sugeno FISs behave for the calibration of traffic models at upper levels of modeling, such as mesoscopic and macroscopic.

Future research can also address the limitations of this research described at the end of Section 6. It is worth analyzing the impact of the use of combinations of different types of membership function (i.e., Gaussian, triangular, trapezoidal, etc.) on the calibration of car-following models. Also, changes can be made in the intervals associated with the membership functions, and the compensation values obtained can be monitored.

The use of fault injection mechanisms can be replaced by approximated values of the possible measurement errors calculated according to the worst-case scenario by subtracting the values corresponding to the tolerance associated with the sensors used for traffic data acquisition. Currently, the data used in this research are provided by the local traffic management center, and the obtained data do not have details related to the data sheets of the inductive loops placed in the city for traffic monitoring.

Another way to collect data for the simulation is to use specific simulators and human subjects. In addition to recording the traffic profiles from the simulator, the emotional behavior of the human subject can also be recorded. The use of tools such as eye-tracking, galvanic skin response analysis, or heart rate analysis facilitates the classification of driver behavior that can be introduced as a third input variable of the FIS. This is essential for adopting the appropriate car-following strategy in the case of autonomous vehicles due to the possible interaction with human-driven vehicles on the road.

## Figures and Tables

**Table 1:** Quantitative scientific literature overview based on database search results.

Scientific Database	Query	Results
ISI Web of Science (WoS)	(“car-following” OR “vehicle-following”) AND “calibration” AND “fuzzy” (Topic)	11
Scopus	TITLE-ABS-KEY ((“car-following” OR “vehicle-following”)AND “calibration” AND “fuzzy”)	10
Google Scholar	(“car-following” OR “vehicle-following”) AND “calibration” AND “fuzzy”	212
	Total	233

**Table 2:** Linguistic variables for the input of the fuzzy inference systems (FISs).

Variable Name	Membership Function Name	Range	Membership Function Type
RELATIVE_VELOCITY	NB	[−25;0]	trapezoidal
	M	[−4.50;4.50]	triangular
	PB	[0;25]	trapezoidal
INTERVEHICLE_SPACING	NB	[−30;0]	trapezoidal
	ZE	[−6.75;6.75]	triangular
	PS	[0;9]	trapezoidal
	PB	[9;30]	trapezoidal

**Table 3:** Fuzzy rules for the inputs and output variables of the fuzzy inference systems (FISs).

	INTERVEHICLE_SPACING			
RELATIVE_VELOCITY	NB	ZE	PS	PB
NB	PB	PS	ZE	NS
ZE	PS	ZE	NS	NB
PB	ZE	NS	NB	NB

**Table 4:** Linguistic variables for Mamdani fuzzy inference systems (FISs).

Variable Name	Membership Function Name	Range	Membership Function Type
s_OFFSET_M	NB	[−25.50;−4.50]	trapezoidal
	NS	[−9;0]	triangular
	ZE	[−4.50;4.50]	triangular
	PS	[0;9]	triangular
	PB	[4.50;25.50]	trapezoidal

**Table 5:** Comparison between results error in the running distance $x_{4}$ of the follower vehicle (FV).

Fuzzy Inference System	MAE	RMSE
Mamdani	9.3816	11.6682
Takagi–Sugeno	2.5408	3.1904

**Table 6:** Comparison between results error in the dynamic safety distance *y*.

Fuzzy Inference System	MAE	RMSE
Mamdani	9.7868	11.6840
Takagi–Sugeno	2.9459	3.3012

## Data Availability

Not applicable.

## References

[1] Shang M., Rosenblad B., Stern R. (2022). A Novel Asymmetric Car Following Model for Driver-Assist Enabled Vehicle Dynamics. IEEE Trans. Intell. Transport. Syst..

[2] Xiao L., Wang M., Van Arem B. (2017). Realistic Car-Following Models for Microscopic Simulation of Adaptive and Cooperative Adaptive Cruise Control Vehicles. Transp. Res. Rec..

[3] Desjardins C., Chaib-draa B. (2011). Cooperative Adaptive Cruise Control: A Reinforcement Learning Approach. IEEE Trans. Intell. Transport. Syst..

[4] Kim B., Heaslip K.P. (2023). Identifying Suitable Car-Following Models to Simulate Automated Vehicles on Highways. Int. J. Transp. Sci. Technol..

[5] Ahmed H.U., Huang Y., Lu P. (2021). A Review of Car-Following Models and Modeling Tools for Human and Autonomous-Ready Driving Behaviors in Micro-Simulation. Smart Cities.

[6] Mamdani E.H., Assilian S. (1975). An Experiment in Linguistic Synthesis with a Fuzzy Logic Controller. Int. J. Man Mach. Stud..

[7] Takagi T., Sugeno M. (1985). Fuzzy Identification of Systems and Its Applications to Modeling and Control. IEEE Trans. Syst. Man Cybern..

[8] Fernandez S., Ito T., Cruz-Piris L., Marsa-Maestre I. (2022). Fuzzy Ontology-Based System for Driver Behavior Classification. Sensors.

[9] Bennajeh A., Ben Said L. (2021). Driving Control Based on Bilevel Optimization and Fuzzy Logic. Int. J. Intell. Syst..

[10] Chakroborty P., Kikuchi S. (2003). Calibrating the Membership Functions of the Fuzzy Inference System: Instantiated by Car-Following Data. Transp. Res. Part Emerg. Technol..

[11] Chen D., Yuan Y., Li B., Wu J., Wang L., Jin Y. (2005). Validation and Comparison of Microscopic Car-Following Models Using Beijing Traffic Flow Data. Proceedings of the Fuzzy Systems and Knowledge Discovery.

[12] Nadimi N., Behbahani H., Shahbazi H. (2016). Calibration and Validation of a New Time-Based Surrogate Safety Measure Using Fuzzy Inference System. J. Traffic Transp. Eng..

[13] Pop M.-D., Proștean O., David T.-M., Proștean G. (2020). Hybrid Solution Combining Kalman Filtering with Takagi–Sugeno Fuzzy Inference System for Online Car-Following Model Calibration. Sensors.

[14] Pop M.-D., Prostean O., Micea M.V. (2022). Evaluation of the Use of an Intelligent System in the Calibration of a Refined Car-Following Model. Proceedings of the 2022 IEEE 22nd International Symposium on Computational Intelligence and Informatics and 8th IEEE International Conference on Recent Achievements in Mechatronics, Automation, Computer Science and Robotics (CINTI-MACRo).

[15] Sun P., Wang X., Zhu M. (2021). Modeling Car-Following Behavior on Freeways Considering Driving Style. J. Transp. Eng. Part A Syst..

[16] Pérez J., Seco F., Milanés V., Jiménez A., Díaz J.C., De Pedro T. (2010). An RFID-Based Intelligent Vehicle Speed Controller Using Active Traffic Signals. Sensors.

[17] Terán J., Navarro L., Quintero M C.G., Pardo M. (2020). Intelligent Driving Assistant Based on Road Accident Risk Map Analysis and Vehicle Telemetry. Sensors.

[18] Ronquillo-Cana C.J., Pancardo P., Silva M., Hernández-Nolasco J.A., Garcia-Constantino M. (2022). Fuzzy System to Assess Dangerous Driving: A Multidisciplinary Approach. Sensors.

[19] Dos Reis Junior J.V., Raddo T.R., Sanches A.L., Borges B.-H.V. Comparison between Mamdani and Sugeno Fuzzy Inference Systems for the Mitigation of Environmental Temperature Variations in OCDMA-PONs. Proceedings of the 2015 17th International Conference on Transparent Optical Networks (ICTON).

[20] Egaji O.A., Griffiths A., Hasan M.S., Yu H.-N. (2015). A Comparison of Mamdani and Sugeno Fuzzy Based Packet Scheduler for MANET with a Realistic Wireless Propagation Model. Int. J. Autom. Comput..

[21] Gupta S., Biswas P.K., Aljafari B., Thanikanti S.B., Das S.K. (2023). Modelling, Simulation and Performance Comparison of Different Membership Functions Based Fuzzy Logic Control for an Active Magnetic Bearing System. J. Eng..

[22] Wang Y., Chen Y. A Comparison of Mamdani and Sugeno Fuzzy Inference Systems for Chaotic Time Series Prediction. Proceedings of the 2012 2nd International Conference on Computer Science and Network Technology.

[23] Hamam A., Georganas N.D. A Comparison of Mamdani and Sugeno Fuzzy Inference Systems for Evaluating the Quality of Experience of Hapto-Audio-Visual Applications. Proceedings of the 2008 IEEE International Workshop on Haptic Audio visual Environments and Games.

[24] Özger M. (2009). Comparison of Fuzzy Inference Systems for Streamflow Prediction. Hydrol. Sci. J..

[25] Phunpeng V., Kerdphol T. (2021). Comparative Study of Sugeno and Mamdani Fuzzy Inference Systems for Virtual Inertia Emulation. Proceedings of the 2021 IEEE PES/IAS PowerAfrica.

[26] Samavat T., Nazari M., Ghalehnoie M., Nasab M.A., Zand M., Sanjeevikumar P., Khan B. (2023). A Comparative Analysis of the Mamdani and Sugeno Fuzzy Inference Systems for MPPT of an Islanded PV System. Int. J. Energy Res..

[27] Boutaybi M., Khlifi Y., Benslimane A., Elhafyani M.L. Optimization of Photovoltaic System Using Mamdani and Takagi Sugeno MPPT Controls. Proceedings of the 2022 2nd International Conference on Innovative Research in Applied Science, Engineering and Technology (IRASET).

[28] Khosravanian R., Sabah M., Wood D.A., Shahryari A. (2016). Weight on Drill Bit Prediction Models: Sugeno-Type and Mamdani-Type Fuzzy Inference Systems Compared. J. Nat. Gas Sci. Eng..

[29] Bagis A., Konar M. (2016). Comparison of Sugeno and Mamdani Fuzzy Models Optimized by Artificial Bee Colony Algorithm for Nonlinear System Modelling. Trans. Inst. Meas. Control..

[30] Saleh I., Alhady S.S.N., Rahiman W., Ibrahim H., Iqbal S., Teoh S.S., Mustaffa M.T. (2017). Comparison of Mamdani and Sugeno Fuzzy Logic Performance as Speed Controller. 9th International Conference on Robotic, Vision, Signal Processing and Power Applications.

[31] Wang Y., Chen Y. (2014). A Comparison of Mamdani and Sugeno Fuzzy Inference Systems for Traffic Flow Prediction. J. Comput..

[32] Saleh A., Fujiati, Rosnelly R., Puspita K., Sanjaya A. A Comparison of Mamdani and Sugeno Method for Optimization Prediction of Traffic Noise Levels. Proceedings of the 2017 5th International Conference on Cyber and IT Service Management (CITSM).

[33] Pan D., Zheng Y. Optimal Control and Discrete Time-Delay Model of Car Following. Proceedings of the 2008 7th World Congress on Intelligent Control and Automation.

[34] Khodayari A., Kazemi R., Ghaffari A., Manavizadeh N. Modeling and Intelligent Control Design of Car Following Behavior in Real Traffic Flow. Proceedings of the 2010 IEEE Conference on Cybernetics and Intelligent Systems.

[35] Zadeh L.A. (1965). Fuzzy Sets. Inf. Control.

[36] Pop M.-D., Proștean O., Proștean G. (2020). Fault Detection Based on Parity Equations in Multiple Lane Road Car-Following Models Using Bayesian Lane Change Estimation. J. Sens. Actuator Netw..

[37] Yin B., Dridi M., El Moudni A. Adaptive Traffic Signal Control for Multi-Intersection Based on Microscopic Model. Proceedings of the 2015 IEEE 27th International Conference on Tools with Artificial Intelligence (ICTAI).

[38] Punzo V., Formisano D.J., Torrieri V. (2005). Nonstationary Kalman Filter for Estimation of Accurate and Consistent Car-Following Data. Transp. Res. Rec..

[39] Li G., Zhu W. (2019). The Car-Following Model Based on Fuzzy Inference Controller. IOP Conf. Ser. Mater. Sci. Eng..

[40] Vassilyev S.N., Kudinov Y.I., Pashchenko F.F., Durgaryan I.S., Kelina A.Y., Kudinov I.Y., Pashchenko A.F. (2020). Intelligent Control Systems and Fuzzy Controllers. I. Fuzzy Models, Logical-Linguistic and Analytical Regulators. Autom. Remote Control.

[41] Barraza M., Matía F., Al-Hadithi B.M. (2023). Dynamic Analysis of Fuzzy Systems. Appl. Sci..

[42] Jassbi J., Alavi S.H., Serra P.J.A., Ribeiro R.A. Transformation of a Mamdani FIS to First Order Sugeno FIS. Proceedings of the 2007 IEEE International Fuzzy Systems Conference.

[43] Mogharreban N., DiLalla L.F. Comparison of Defuzzification Techniques for Analysis of Non-Interval Data. Proceedings of the NAFIPS 2006–2006 Annual Meeting of the North American Fuzzy Information Processing Society.

[44] Bennajeh A., Bechikh S., Said L.B., Aknine S. (2019). Bi-Level Decision-Making Modeling for an Autonomous Driver Agent: Application in the Car-Following Driving Behavior. Appl. Artif. Intell..

[45] Punzo V., Simonelli F. (2005). Analysis and Comparison of Microscopic Traffic Flow Models with Real Traffic Microscopic Data. Transp. Res. Rec..

[46] Zhou Y., Fei R. A Novel Car-Following Model by Considering Driver’s Behaviors with Fuzzy Inference Method. Proceedings of the 2021 3rd International Symposium on Smart and Healthy Cities (ISHC).

[47] Ross T.J. (2010). Fuzzy Logic with Engineering Applications.

